# Textural Analysis of the Hyperdense Artery Sign in Patients with Acute Ischemic Stroke Predicts the Outcome of Thrombectomy

**DOI:** 10.3390/jcdd10090359

**Published:** 2023-08-24

**Authors:** Lucian Mărginean, Rares Cristian Filep, Bogdan Andrei Suciu, Tudor G. Jovin, Paul-Andrei Ștefan, Roxana-Adelina Lupean, Eliza Mihaela Arbănași, Emil Marian Arbănași, Diana Roxana Opriș, Alexander Niklas Timm, Rareș Vodă, Vlad Vunvulea

**Affiliations:** 1 Radiology and Medical Imaging, Clinical Sciences Department, “George Emil Palade” University of Medicine, Pharmacy, Science and Technology of Targu Mures, 540139 Targu Mures, Romania; lucian.marginean@umfst.ro; 2 Interventional Radiology Department, Târgu Mureș County Emergency Clinical Hospital, 540136 Targu Mures, Romania; 3 Department of Anatomy, “George Emil Palade” University of Medicine, Pharmacy, Science and Technology of Targu Mures, 540139 Targu Mures, Romania; 4 Cooper Neurological Institute, Cherry Hill, NJ 08002, USA; jovin-tudor@cooperhealth.edu; 5 Anatomy and Embryology, Morphological Sciences Department, “Iuliu Haţieganu” University of Medicine and Pharmacy, Victor Babeș, 400012 Cluj-Napoca, Romania; 6 Radiology and Imaging Department, County Emergency Hospital, Clinicilor Street, Number 3–5, 400006 Cluj-Napoca, Romania; 7 Histology, Morphological Sciences Department, “Iuliu Hațieganu” University of Medicine and Pharmacy, Louis Pasteur Street, Number 4, 400349 Cluj-Napoca, Romania; roxanalupean92@gmail.com; 8 Faculty of Pharmacy, “George Emil Palade” University of Medicine, Pharmacy, Science and Technology of Targu Mures, 540139 Targu Mures, Romania; 9 Clinic of Vascular Surgery, Mures County Emergency Hospital, 540136 Targu Mures, Romania; 10 Center for Advanced Medical and Pharmaceutical Research, “George Emil Palade” University of Medicine, Pharmacy, Science and Technology of Targu Mures, 540139 Targu Mures, Romania; 11 Emergency Institute for Cardiovascular Diseases and Transplantation (IUBCVT) of Târgu Mureș, 540136 Targu Mures, Romania; 12 Radiology and Medical Imaging Laboratory, Târgu Mureș County Emergency Clinical Hospital, 540136 Targu Mures, Romania

**Keywords:** acute ischemic stroke, large vessel occlusion, endovascular, thrombectomy, aspiration, stent-retriever, prognosis, radiomics, textural features

## Abstract

Textural analysis is pivotal in augmenting the diagnosis and outcomes of endovascular procedures for stroke patients. Due to the detection of changes imperceptible to the human eye, this type of analysis can potentially aid in deciding the optimal type of endovascular treatment. We included 40 patients who suffered from acute ischemic stroke caused by large vessel occlusion, and calculated 130 different textural features based on the non-enhanced CT scan using an open-source software (3D Slicer). Using chi-squared and Mann–Whitney tests and receiver operating characteristics analysis, we identified a total of 21 different textural parameters capable of predicting the outcome of thrombectomy (quantified as the mTICI score), with variable sensitivity (50–97.9%) and specificity (64.6–99.4%) rates. In conclusion, CT-based radiomics features are potential factors that can predict the outcome of thrombectomy in patients suffering from acute ischemic stroke, aiding in the decision between aspiration, mechanical, or combined thrombectomy procedure.

## 1. Introduction

Stroke, a prevalent cerebrovascular disease, constitutes a substantial portion of global morbidity and mortality. It is marked by the abrupt loss of brain function due to a disturbance in blood supply to the brain, typically caused by ischemia, haemorrhage, or embolism [1].

In Europe, stroke is the second leading cause of death after ischemic heart disease and a principal source of adult disability according to the World Health Organization. Approximately 1.5 million strokes occur annually in Europe, highlighting its significant disease burden according to the 2020 report of the European Stroke Organization. The incidence of stroke differs substantially among European countries, with Eastern Europe reporting higher rates compared to Western Europe [2].

The diagnosis of stroke is primarily clinical, but neuroimaging techniques are an integral part of the confirmation of the diagnosis and can help to distinguish between ischemic and hemorrhagic stroke entities [3]. Computed tomography (CT) scans both non-enhanced and enhanced by contrast substance are often the first imaging modality used due to their wide availability, rapid scanning time, and reliability in ruling out intracranial hemorrhage. Magnetic Resonance Imaging (MRI) is an alternative which while more sensitive for the detection of ischemic stroke in the early hours, is less accessible and takes longer to perform [4].

Contrast-enhanced CT (CECT) and perfusion CT are invaluable imaging techniques in the field of neuroimaging, playing a crucial role in the assessment and management of various stroke cases, particularly those with extended time windows, unknown onset, and wake-up stroke scenarios. These advanced imaging modalities have revolutionized the diagnostic landscape by providing clinicians with a comprehensive understanding of cerebral perfusion and vascular patency [5].

In cases of extended-time-window strokes, where the onset of symptoms exceeds the conventional thrombolytic time window, CECT and perfusion CT allow for a more precise evaluation of the ischemic penumbra and infarct core. This information aids in identifying potentially salvageable brain tissue, thus enabling better decision-making regarding potential interventions such as thrombectomy or other neuroprotective strategies [6].

In scenarios where the exact onset of symptoms is unknown, CECT and perfusion CT play a critical role in distinguishing acute from chronic infarcts, guiding treatment strategies, and mitigating the risks associated with delayed intervention. The ability to assess perfusion deficits and identify areas of active ischemia significantly impacts the therapeutic approach and enhances patient outcomes [7].

In the management of acute ischemic stroke, endovascular treatment, particularly mechanical thrombectomy, has increasingly become the gold standard, demonstrating superior efficacy to traditional approaches in certain patients. This therapeutic paradigm shift was driven by several landmark trials, including MR CLEAN, ESCAPE, and DEFUSE 3, which unequivocally evidenced the superiority of endovascular thrombectomy over intravenous thrombolysis alone for large vessel occlusions [7,8,9]. Particularly, DEFUSE 3 extended the window for thrombectomy to 16–24 h, provided the existence of salvageable brain tissue on advanced imaging. In contrast to intravenous thrombolysis, which is limited by a narrower therapeutic window and a risk of intracranial hemorrhage, endovascular treatment offers a higher recanalization rate and improved functional outcomes, substantially enhancing the prospect of recovery in acute ischemic stroke patients [10].

The utilization of non-enhanced computed tomography (NECT) scans in stroke diagnosis has intrinsic limitations that impede an exhaustive assessment [11]. Primarily, NECT scans can be insensitive to early ischemic changes, and distinguishing between ischemic penumbra and irreversibly infarcted tissue can be challenging. This curtails the capacity to accurately gauge the extent of cerebral infarction. Moreover, the visualization of thrombi, particularly if they are small or located in the posterior circulation, can be suboptimal in NECT [5]. To address these drawbacks, textural analysis and radiomics have emerged as paramount tools. By employing sophisticated algorithms to quantitatively analyse the textural features of images, such as gray-level intensity, homogeneity, and variations, it becomes possible to extract clinically relevant information that is imperceptible to the human eye. The heterogeneity of a thrombus, as discerned through textural analysis, can have prognostic implications [12]. For instance, textural heterogeneity may indicate variations in thrombus composition, which could influence the response to thrombolytic therapy. Additionally, radiomics can be harnessed for predictive modelling, where the extracted features are correlated with clinical outcomes. This enhances the prognostic stratification and helps tailor patient-specific interventions, thereby optimizing stroke management [13].

The purpose of this paper is to present the associations between the radiomic features in non-enhanced CT scans and the outcome of the endovascular treatment in stroke patients caused by acute large vessel occlusion (LVO).

## 2. Materials and Methods

### 2.1. Study Design

This Health Insurance Portability and Accountability Act–compliant, single-institution, retrospective pilot-study has been approved by the institutional review board (Ethical Committee of the Mureș County Emergency Hospital; No. 14527/15.06.2016). Written informed consent was provided by all subjects, owing to the retrospective nature of this research.

We designed our study to be a retrospective, analytical cohort study where we included all patients presented to the Interventional Radiology Compartment of the Mures County Emergency Hospital, with the diagnosis of acute ischemic stroke. All patients included in our study were over the age of 18, the time period of interest being January 2022–January 2023. All patients presented to our compartment presented neurological and imagistic signs of stroke, confirmed by a native and contrast-enhanced CT scan. We accepted patients with an ASPECTS score >7, evaluated by an independent radiologist.

From a total of 135 patients referred to our Clinic, we excluded patients who presented hemorrhagic transformation of the ischemic stroke (*n* = 36), patients for whom selective catheterization of the supra-aortic vessels was not possible (*n* = 7), patients with spontaneous recanalization (*n* = 17), and patients for whom the initial CT scan did not indicate the “hyperdense artery” sign (*n* = 35). This latter criteria for exclusion is crucial, since the textural analysis relies on the presence of a hyperdense thrombus on the native CT scan. Furthermore, in patients with ischemic stroke who present hemorrhagic transformation, the endovascular treatment is contraindicated. In the context of the conducted study, it is imperative to state that there were no patients enrolled who exhibited atrial fibrillation or had undergone prosthetic valve implantation at the time of stroke onset.

### 2.2. Data Collection

All examinations were reviewed by one radiologist (PAȘ, with 4 years of experience in stroke imaging), who was also aware of the clinical data and the patients’ final diagnosis, in order to assure the quality of the selected data. The patients’ medical information was retrieved from the archive of our healthcare unit. After this step, the researcher (PAS) was excluded from the image analysis process and the statistical analysis process.

Considering that the majority of patients were sent from nearby hospitals, we imported the native CT scan of each patient into the hospital Picture archiving and communication system (PACS) database, for further analysis and segmentation. We also collected the following data from our patients: age, sex, medical history of diabetes, arterial hypertension, tobacco use. We were also interested in the following parameters of the endovascular treatment: modified treatment in cerebral ischemia (mTICI) score, type of intervention (aspiration thrombectomy, stent-retriever assisted thrombectomy, combined approach), type and brand of materials used, and total duration of the intervention.

### 2.3. Textural Analysis

For the segmentation and radiomics feature extraction, we imported the Digital Imaging and Communications in Medicine (DICOM) files of the CT scans into 3D Slicer (a commercially available software at www.slicer.org (accessed on 20 November 2022)) [14].

The precise methodology included the following steps: (1) To accomplish image normalization and infarct area segmentation, the boundaries of the infarct zones were defined by modulating the grayscale value of the CT images. Subsequent semi-automatic segmentation yielded the three-dimensional Regions of Interest (ROIs) of the infarct zones. Image voxel resampling to a 1 mm × 1 mm × 1 mm dimension was performed for all ROIs, coupled with Gaussian filter smoothing and fixed image grayscale bin width value at 25 to normalize the images. (2) Extraction of radiomics features was executed from the ROIs of images via the Pyradiomics plugin in the software, covering 3D-shaped features, first-order characteristics, gray-level cooccurrence matrix (GLCM), gray-level dependence matrix (GLDM), gray-level run length matrix (GLRLM), gray-level size zone matrix (GLSZM) and neighboring gray-tone difference matrix (NGTDM). All examinations were reviewed by 1 radiologist doctor with over 25 years of experience in neuroradiology, and 2 resident doctors, all blinded to the final diagnosis.

From a total of 130 radiomic features, we selected the following classes and features:

- First order features: 10Percentile, 90Percentile, Energy, Entropy, InterquartileRange, Kurtosis, Maximum, MeanAbsoluteDeviation, Mean, Median, Minimum, Range, RobustMeanAbsolute Deviation, RootMeanSquared, Skewness, TotalEnergy, Uniformity, Variance
- Gray Level Co-Occurrence Matrix (GLCM) functions: Autocorrelation, ClusterProminence, ClusterShade, ClusterTendency, Contrast, Correlation, DifferenceAverage, DifferenceEntropy, DifferenceVariance, Id, Idm, Idmn, Idn, Imc1, Imc2, InverseVariance, JointAverage, JointEnergy, JointEntropy, MCC, MaximumProbability, SumAverage, SumEntropy, SumSquares
- Gray Level Dependence Matrix (GLDM) features: DependenceEntropy, DependenceNonUniformity, DependenceNonUniformityNormalized, DependenceVariance, GrayLevelNonUniformity, GrayLevelVariance, HighGrayLevelEmphasis, LargeDependenceEmphasis, LargeDependenceHighGrayLevelEmphasis, LargeDependenceLowGrayLevelEmphasis, LowGrayLevelEmphasis, SmallDependenceEmphasis, SmallDependenceHighGrayLevelEmphasis, SmallDependenceLowGrayLevelEmphasis
- Gray Level Run Length Matrix (GLRLM) Features: GrayLevelNonUniformity, GrayLevelNonUniformityNormalized, GrayLevelVariance, HighGrayLevelRunEmphasis, LongRunEmphasis, LongRunHighGrayLevelEmphasis, LongRunLowGrayLevelEmphasis, LowGrayLevelRunEmphasis, RunEntropy, RunLengthNonUniformity, RunLengthNonUniformityNormalized, RunPercentage, RunVariance, ShortRunEmphasis, ShortRunHighGrayLevelEmphasis, ShortRunLowGrayLevelEmphasis
- Gray Level Size Zone Matrix (GLSZM) Features: GrayLevelNonUniformity, GrayLevelNonUniformityNormalized, GrayLevelVariance, HighGrayLevelZoneEmphasis, LargeAreaEmphasis, LargeAreaHighGrayLevelEmphasis, LargeAreaLowGrayLevelEmphasis, LowGrayLevelZoneEmphasis, SizeZoneNonUniformity, SizeZoneNonUniformityNormalized, SmallAreaEmphasis, SmallAreaHighGrayLevelEmphasis, SmallAreaLowGrayLevelEmphasis, ZoneEntropy, ZonePercentage, ZoneVariance.
- Neighbouring Gray Tone Difference Matrix (NGTDM) Features: Busyness, Coarseness, Complexity, Contrast, Strength.

### 2.4. Statistical Analysis

For the statistical analysis, we used the software SPSS for Mac OS version 28.0.1.0 (SPSS, Inc., Chicago, IL, USA). The ROC curve analysis was used to determine the appropriate procedure duration and several textural features cut-off values based on the Youden index (Youden Index = Sensitivity + Specificity − 1, ranging from 0 to 1) for each monitored outcome. To evaluate the associations of textural features with the category factors, we used chi-square tests, and for continuous variables, we used the Mann–Whitney test.

### 2.5. Study Outcomes

The main objective of this study is to investigate the potential association between radiomics features extracted from CT scans and the clinical outcome of endovascular thrombectomy procedures (quantified as a mTICI > 2b) in patients with acute ischemic stroke. Specifically, we aim to assess whether certain radiomics features present in the hyperdense artery sign, as observed in CT scans, can serve as predictive markers for the success or failure of endovascular interventions.

As a secondary objective, this study seeks to identify specific radiomics features that are independently associated with favorable outcomes following different types of endovascular thrombectomy procedures, including aspiration thrombectomy, mechanical thrombectomy, and combined thrombectomy approaches. The analysis will focus on determining which radiomics characteristics exhibit a statistically significant correlation with improved outcomes (mTICI > 2b) for each respective thrombectomy technique.

## 3. Results

During the examined period, we performed 135 endovascular curative procedures for stroke patients. Out if these patients, we identified a total of 40 patients suffering from acute ischemic stroke caused by large vessel occlusion, with the hyperdense artery sign visible on the native head CT scan, that met our inclusion criteria.

We initially split the patients into two categories, depending on the outcome of the endovascular procedure, using the modified TICI score [15], the first lot being patients with favorable outcome (mTICI ≥ 2b), the second lot consisting in patients with unfavorable outcome (mTICI < 2b). Furthermore, we investigated the possible associations between the radiomical features of the thrombus on the native CT scan and the outcome of the patients after endovascular treatment. We calculated all 130 radiomical features for our patients, and continued to the presentation of only statistically significant associations.

In Table 1, we present the statistically significant associations between the textural features and the outcome of all endovascular procedures (aspiration thrombectomy, mechanical thrombectomy, and combined aspiration-mechanical thrombectomy).

**Figure 1:**
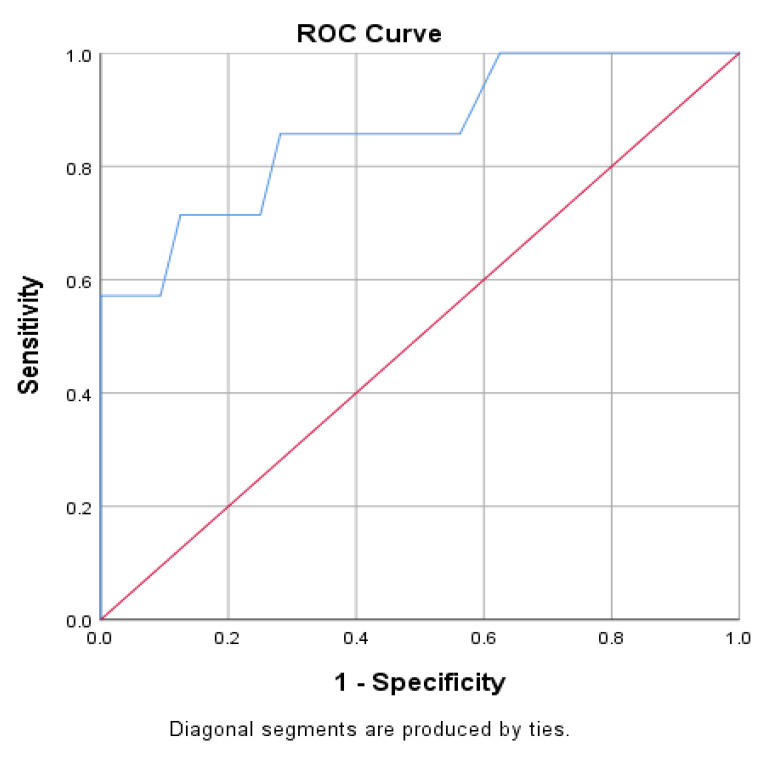
Graphical representation of the ROC curve of the association between procedure duration and outcome, the blue line signifies the procedure duration, the red line signifies the random classifier.

We then computed the receiver operating characteristic (ROC) curve in Figure 1, and present the ROC analysis in Table 2.

Furthermore, we selected only patients who underwent only aspiration thrombectomy and compared the radiomical features against the outcomes of the endovascular procedure. We present our results in Table 3.

**Figure 2:**
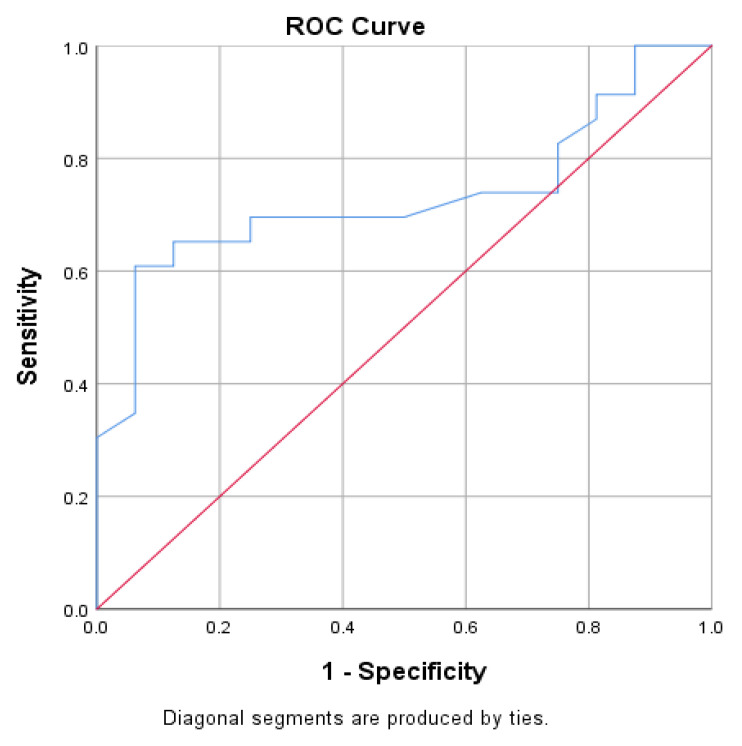
Graphical representation of the ROC curve of the association between procedure duration and outcome the blue line signifies the procedure duration, the red line signifies the random classifier.

We then computed the receiver operating characteristic (ROC) curve in Figure 2, and present the ROC analysis in Table 4.

We then proceeded to select patients who benefited from mechanical thrombectomy alone as endovascular treatment of acute ischemic stroke. The associations between the textural features and the outcome of the mechanical thrombectomy are represented in Table 5.

**Figure 3:**
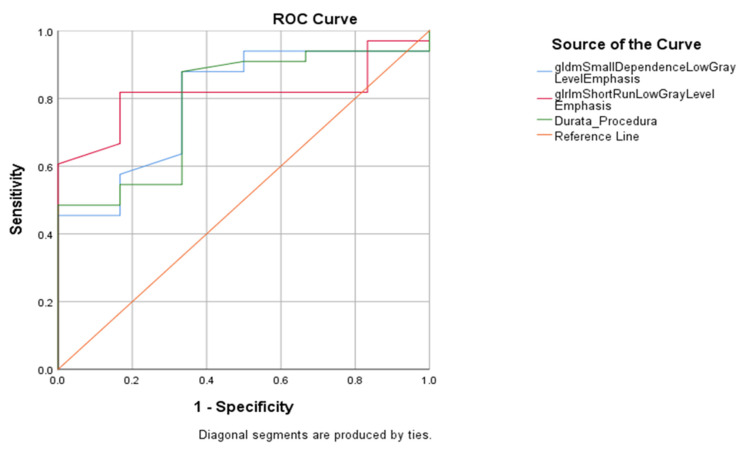
Graphical representation of the ROC curve of the association between textural features, procedure duration, and outcome of mechanical thrombectomy procedure.

**Figure 4:**
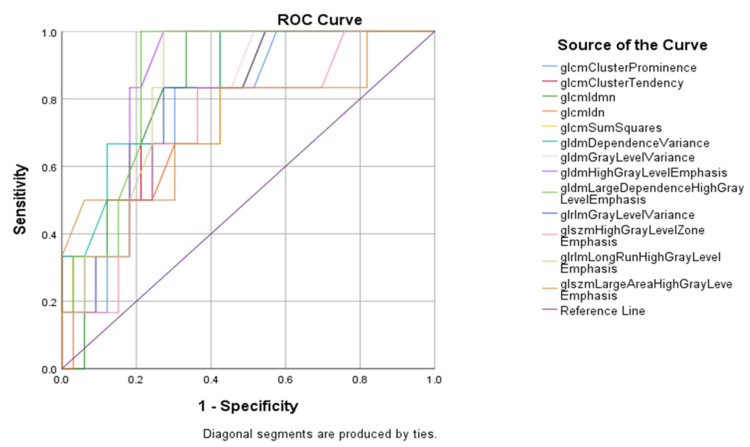
Graphical representation of the ROC curve of the association between textural features and outcome of mechanical thrombectomy procedure.

Furthermore we present the results of the ROC curve for the analyzed parameters in patients who received mechanical thrombectomy alone in Figure 3 and Figure 4 and Table 6.

**Figure 5:**
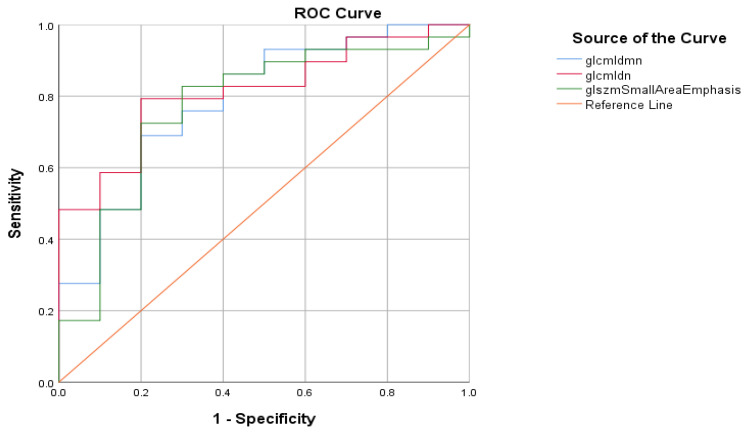
Graphical representation of the ROC curve of the association between textural features and outcome of combined thrombectomy procedure.

We also proceeded to analyse the associations between the textural features on the CT scan and the outcome of endovascular procedures in which a combination of simultaneous mechanical and aspiration thrombectomy was performed—Solumbra technique. We present the results in Table 7. Figure 5 and Table 8 contain the ROC curve and the analysis result.

## 4. Discussion

Our results show that the procedure duration is associated with the outcome of thrombectomy in patients with acute ischemic stroke, regardless of the type of procedure. Furthermore, we identified GrayLevelVarience (*p* = 0.028), a GLRLM feature, to be associated with the favorable outcome (mTICI ≥ 2b) of patients, regardless of the type of procedure.

For patients on whom aspiration thrombectomy was performed, we found ClusterShade (*p* = 0.04) and SizeZoneNonUniformity (*p* = 0.037) to be significantly associated with the outcome of the procedure.

The highest number of textural features were associated with the outcome of mechanical thrombectomy, a total of 15 features: ClusterProminence (*p* = 0.019), ClusterTendency (*p* = 0.015), Idmn (*p* = 0.049), Idn (*p* = 0.05), SumSquares (*p* = 0.016), DependenceVariance (*p* = 0.004), GrayLevelVariance-GLDM (*p* = 0.019), HighGrayLevelEmphasis (*p* = 0.024), LargeDependenceHighGrayLevelEmphasis (*p* = 0.007), SmallDependenceLowGrayLevelEmphasis (*p* = 0.039), GrayLevelVariance-GLRLM (*p* = 0.026), HighGrayLevelRunEmphasis (*p* = 0.022), LongRunHighGrayLevelEmphasis (*p* = 0.011), ShortRunHighGrayLevelEmphasis (*p* = 0.026), ShortRunLowGrayLevelEmphasis (*p* = 0.043), and LargeAreaHighGrayLevelEmphasis (*p* = 0.014). As for patients who received a combined thrombectomy approach (both aspiration and mechanical thrombectomy in the same session), we identified ldmn (*p* < 0.001), ldm (*p* = 0.004) and SmallAreaEmphasis (*p* = 0.02) to be statistically associated with the outcome of the endovascular procedure.

The role of textural analysis in the prediction of various pathologies has lately become a trending topic for various studies [16,17,18,19,20,21]. These features provide quantitative measures of the spatial distribution of pixel intensities within the brain tissue, offering valuable insights into the underlying pathophysiology of stroke and its response to treatment. By leveraging radiomics analysis and advanced machine learning algorithms, researchers have sought to identify specific textural features that can aid in treatment decision-making, improve patient selection for thrombectomy procedures, and ultimately enhance stroke outcomes.

The outcome of thrombectomy can vary among patients, and it is crucial to identify factors that can predict the success or failure of the procedure. In recent years, there has been growing interest in studying the textural features of thrombi on native CT scans and their potential power to predict the outcome of thrombectomy [6,22,23].

A study conducted by Hofmeister et. al. [12] proved that there is a relationship between the textural parameters and the biochemical composition of the clots responsible for the AIS, and the outcome of the patient. According to their study 9 parameters belonging to the GLCM and GLDM group were associated with the procedure outcome. The findings of the present study validate the findings of Hofmeister et. al. [12], with the confirmation that LargeAreaHighGrayLevelEmphasis is a predictive factor in the outcome of mechanical thrombectomy procedure (*p* = 0.037, AUC = 0.737, Sensitivity 50%, Specificity 99.4%).

A different study conducted by Xiong et. al. [13] on the radiomics features of thrombi in AIS patients proves that some features have a significant predictive power in the outcome of thrombectomy procedures. Out of all the parameters determined in their study, we identify ShortRunLowGrayLevelEmphasis (*p* = 0.0016, AUC = 0.813, Sensitivity 81.8%, Specificity 83.3%) and LargeAreaHighGrayLevelEmphasis (*p* = 0.037, AUC = 0.737, Sensitivity 50%, Specificity 99.4%) as predictors for the outcome of mechanical thrombectomy procedures. Various other radiomics features identified as prognostic factors may be explained by the fact that machine learning and a validation cohort were used for the deep learning algorithms.

Yusuying et al. [24] conducted a research study which aimed to examine the efficacy of a computed tomography (CT)-based thrombus Radiomics normogram in predicting the occurrence of secondary embolization during mechanical thrombectomy (MT) in cases of large vessel occlusion. The study involved a training cohort, validation cohort, and the use of eight machine learning algorithms trained and subsequently employed for the purpose of Radiomics feature analysis. The findings of the study demonstrate that the proposed normogram holds great potential as a novel predictive model for assessing the likelihood of secondary embolization during interventional procedures in the management of acute ischemic stroke. A novel clinical Radiomics model was suggested for the anticipation of secondary embolization. The model incorporates a fusion of Radiomics and clinical features, which demonstrated superior predictive accuracy compared to using Radiomics features or clinical features independently. Some of the findings in the study of Yusuying et al. [24] are similar to our findings, such as the predictive power of LongRunHighGrayLevelEmphasis (AUC = 0.838, *p* = 0.009, Sensitivity 83.3%, Specificity 77.7%).

A different study conducted by Li et al. [25] evaluated the prognostic value of textural features based on the hyperdense artery sign on native CT scans in patients with AIS. The investigators created Phyton scripts for logistic regression models in order to determine valid prediction models. They identified 8 significant textural features associated with the outcome of thrombectomy in stroke patients, the most promising one being GrayLevelVariance. Similar to the study conducted by Li et al. [25], we also identified GrayLeveVariance (AUC = 0.783, *p* = 0.029, Sensitivity 83.3%, Specificity 73.7%) as a prognostic factor for the outcome of mechanical thrombectomy in patients suffering from acute ischemic stroke. Similar to the aforementioned study, we also identified SmallAreaEmphasis (AUC = 0.772, *p* = 0.011, Sensitivity 82.8%, Specificity 70%) as prediction factors for the outcome of AIS patients on whom a combined aspiration and mechanical thrombectomy was performed.

The findings of our study validate the results of other recent research from the last 4 years. We consider our work to be particularly relevant since we also split the patients by procedure type: aspiration thrombectomy, mechanical thrombectomy, and combined thrombectomy. The optimal approach from an endovascular standpoint in the treatment of acute ischemic stroke caused by large vessel occlusion is still debated in the literature.

Several studies have contributed to the debate by comparing the efficacy and safety of these different approaches. The THRACE study, for example, demonstrated that mechanical thrombectomy combined with intravenous thrombolysis was effective in treating acute ischemic stroke patients [26,27]. The PISTE study also supported the use of endovascular intervention, showing comparable outcomes to mechanical thrombectomy after CT angiography [26,28].

In recent years, there has been a shift towards the use of mechanical thrombectomy as the primary approach. This is supported by studies such as the ANGEL registry, which aimed to evaluate endovascular treatment delivery and improve treatment algorithms in stroke patients [29]. The registry included patients with large-artery occlusion and assessed functional independence as the primary efficacy endpoint.

However, the debate continues as to whether aspiration thrombectomy or combined approaches may offer advantages in certain cases. The GASS Trial, a multicenter study, is currently underway to compare general anesthesia and sedation during intra-arterial treatment for stroke [30].

It is important to note that the choice of technique may depend on various factors, including the location and characteristics of the occlusion, patient-specific factors, and the expertise of the treating team. The decision-making process should involve a multidisciplinary approach, considering the available evidence, individual patient characteristics, and local expertise [26].

The debate surrounding aspiration, mechanical thrombectomy, and combined approaches in endovascular treatment for stroke continues. While mechanical thrombectomy has gained prominence as the primary approach, further research is needed to determine the optimal technique for specific patient populations and occlusion locations. In order to shed some light on this storm of debate, we launched the current study to identify whether the textural features of thrombi on native CT scans can aid the decision-making process regarding which type of endovascular procedure should be used.

Aside from optimizing the outcome of endovascular procedures in AIS, we must also not forget the economic burden of stroke [31]. In a medical system where financing is limited, such as any Romanian Medical Center, the choice of device used for thrombectomy needs to be wise. Using the native CT scan (performed in all patients with stroke) in order to extract textural features that could potentially aid the decision-making process regarding the choice of the optimal device for thrombectomy whilst having almost no financial impact on the medical system. This is one of the motivations for performing the current study. Taking into account the raised interest in prognostic factors for the outcome of endovascular procedures [32,33,34,35,36], we consider our study to be highly significant as it delves into hitherto underexplored dimensions of these factors, facilitating a more comprehensive understanding of their interplay and impact.

In spite of all the benefits of the current study, we have identified a series of limitations of our work. One of the major limitations of our study relies in the design of the study. Being a retrospective single-centre study with a limited sample size, the location of the occluded artery, the variations in anatomy and other comorbidities of patients—factors that can influence the outcome of the procedure—were not taken into account. Extending the study to a multicentric study and separately analysing each separate location of occlusion could potentially overcome these limitations.

Another potential limitation of our study concerns the methods used to construct the current paper. All CT scans were segmented manually. The manual segmentation technique exhibits inherent variability in the selection of threshold adjustments, such as up-scaling or down-scaling, whilst implementing a mask during the segmentation process. In June 2023, Lin et al. [37] conducted a recent study focused on the application of deep learning algorithms for the automation of segmentation and extraction of textural features in hypopharyngeal cancer on MRI. The study observed a significant correlation (*p* < 0.001) between the results obtained from the deep learning algorithm and the manually segmented features. Several deep learning-based mechanism models have demonstrated successful automatic segmentation of carotid plaque, as evidenced by a study conducted by Chen et al. [38] in February 2023. These models, despite lacking a direct association with feature extraction in stroke, have yielded satisfactory results, and could potentially be implemented in future studies in order to homogenize the results across multiple overserves.

The histopathological examination of the blood clot leading to acute ischemic stroke (AIS) was not quantified in our research, making it another limitation of our study. Consequently, it is challenging to establish a direct correlation between textural features and the specific histopathological attributes of the clot responsible for AIS in this particular study. In prospective investigations, it is recommended to incorporate these parameters and conduct an analysis of thrombi with respect to their composition, subsequently facilitating comparisons to establish additional correlations.

Despite all these limitations, we consider this study to be a stepping-stone towards further analysis of textural features and their implications in the outcome of thrombectomy in stroke patients. We consider these factors to be especially important due to their relative ease of use, the favourable economic impact, and their prediction capabilities.

## 5. Conclusions

Our data show that the duration of the thrombectomy procedure is a prognostic factor in the outcome of the procedure in all types of procedures, in situations where aspiration thrombectomy alone was performed, or in situations where mechanical thrombectomy alone was performed. This finding validates the knowledge of the current literature.

We also identified GrayLevelVarience as a predicting factor for favourable outcome in thrombectomy regardless of the procedure type. ClusterShade and SizeZoneNonUniformity are powerful predictors for the outcome of the endovascular procedure where only aspiration thrombectomy was performed. ClusterProminence, ClusterTendency, Idmn, Idn, SumSquares, DependenceVariance, GrayLevelVariance, HighGrayLevelEmphasis, LargeDependenceHighGrayLevelEmphasis, GrayLevelVariance, HighGrayLevelZoneEmphasis, LongRunHighGrayLevelEmphasis, LargeAreaHighGrayLevelEmphasis, SmallDependenceLowGrayLevelEmphasis, and ShortRunLowGrayLevelEmphasis are the textural features that showed association with the outcome of the endovascular procedure where mechanical thrombectomy alone was performed.

Lastly Idmn, Idn, and SmallAreaEmphasis are textural features associated with the outcome of thrombectomy, where both aspiration catheters and stent-retrievers were used in the same session.

We consider the findings regarding textural analysis to be highly significant in an era where machine learning seems to be the future in aiding diagnosis and guiding treatment, especially in stroke patients.

## Figures and Tables

**Table 1:** Statistical analysis of the textural features and procedure duration with the outcome of all thrombectomy procedures.

		Total	mTICI ≥ 2b	mTICI < 2b	*p* Value
GrayLevelVarience	Mean ± Standard deviation	1143.16 ± 7104.37	21.67 ± 47.25	6430.16 ± 16,986.10	0.028
	Lower bound	−1128.93	4.92	−9279.36	
	Upper bound	3415.25	38.43	22,139.69	
Procedure duration (min)	Mean ± Standard deviation	125.7 ± 45.01	35.71 ± 6.22	48.57 ± 18.36	<0.001
	Lower bound	58.08	48.52	80.79	
	Upper bound	86.87	73.84	170.64	

**Table 2:** The receiver operating characteristic analysis results of the association between procedure duration and outcome in all patients.

Parameter	AUC	Sign.lvl.	Cut-Off	Se (%)	Sp (%)
Procedure duration	0.86	0.003	<105	71.4%	87.5%

**Table 3:** Statistical analysis of the textural features and procedure duration with the outcome of only aspiration thrombectomy.

		Mean	Std. Deviation	95% Confidence Interval for Mean		*p* Value
				Lower Bound	Upper Bound	
ClusterShade	mTICI < 2b	16.32	614.20	−249.28	281.92	0.04
	mTICI ≥ 2b	1119.24	2373.64	−101.17	2339.65	
	Total	485.06	1682.00	−52.87	1022.99	
SizeZoneNonUniformity	mTICI < 2b	30.34	65.03	2.22	58.46	0.037
	mTICI ≥ 2b	23.66	32.75	6.82	40.50	
	Total	27.50	53.26	10.47	44.54	
Procedure duration	mTICI < 2b	89.30	50.04	67.67	110.94	<0.001
	mTICI ≥ 2b	49.71	23.18	37.79	61.62	
	Total	72.48	45.01	58.08	86.87	

**Table 4:** The receiver operating characteristic analysis results of the association between procedure duration and outcome of aspiration thrombectomy procedure.

Parameter	AUC	Cut-Off	Se (%)	Sp (%)	*p* Value
Procedure duration	0.73	<74	60.9%	93.75%	0.015

**Table 5:** Statistical analysis of the textural features and procedure duration with the outcome of patients on whom mechanical thrombectomy alone was performed.

		Mean	Std. Deviation	95% Confidence Interval for Mean		*p* Value
				Lower Bound	Upper Bound	
ClusterProminence	mTICI < 2b	31,703.58	82,953.03	2759.90	60,647.26	0.019
	mTICI ≥ 2b	475,355.60	1,106,487.74	−685,832.07	1,636,543.27	
	Total	98,251.38	434,194.62	−40,610.79	237,113.56	
ClusterTendency	mTICI < 2b	34.27	75.85	7.81	60.74	0.015
	mTICI ≥ 2b	302.46	624.90	−353.34	958.26	
	Total	74.50	253.65	−6.62	155.62	
Idmn	mTICI < 2b	0.97	0.02	0.96	0.98	0.049
	mTICI ≥ 2b	0.98	0.01	0.98	0.99	
	Total	0.97	0.02	0.97	0.98	
Idn	mTICI < 2b	0.92	0.03	0.91	0.93	0.050
	mTICI ≥ 2b	0.94	0.02	0.92	0.97	
	Total	0.92	0.03	0.91	0.93	
SumSquares	mTICI < 2b	9.92	21.79	2.32	17.52	0.016
	mTICI ≥ 2b	77.68	158.21	−88.35	243.71	
	Total	20.08	64.89	−0.67	40.84	
DependenceVariance	mTICI < 2b	23.77	7.35	21.20	26.33	0.004
	mTICI ≥ 2b	33.87	7.74	25.75	41.99	
	Total	25.28	8.17	22.67	27.90	
GrayLevelVariance	mTICI < 2b	10.81	23.75	2.53	19.10	0.019
	mTICI ≥ 2b	77.10	156.56	−87.20	241.40	
	Total	20.76	64.76	0.04	41.47	
HighGrayLevelEmphasis	mTICI < 2b	36.09	75.67	9.69	62.50	0.024
	mTICI ≥ 2b	200.23	389.50	−208.52	608.99	
	Total	60.71	166.79	7.37	114.06	
LargeDependenceHighGrayLevelEmphasis	mTICI < 2b	1099.31	991.91	753.21	1445.40	0.007
	mTICI ≥ 2b	9136.05	17,263.47	−8980.85	27,252.95	
	Total	2304.82	6891.11	100.93	4508.70	
SmallDependenceLowGrayLevelEmphasis	mTICI < 2b	0.01	0.01	0.01	0.02	0.039
	mTICI ≥ 2b	0.01	0.00	0.00	0.01	
	Total	0.01	0.01	0.01	0.02	
GrayLevelVariance	mTICI < 2b	11.72	25.84	2.71	20.74	0.026
	mTICI ≥ 2b	64.36	124.89	−66.71	195.42	
	Total	19.62	54.10	2.32	36.92	
HighGrayLevelRunEmphasis	mTICI < 2b	40.46	84.36	11.03	69.90	0.022
	mTICI ≥ 2b	241.72	476.11	−257.93	741.37	
	Total	70.65	200.95	6.39	134.92	
LongRunHighGrayLevelEmphasis	mTICI < 2b	73.18	106.66	35.96	110.40	0.011
	mTICI ≥ 2b	572.93	1131.83	−614.86	1760.71	
	Total	148.14	454.45	2.80	293.48	
ShortRunHighGrayLevelEmphasis	mTICI < 2b	37.48	81.17	9.16	65.80	0.026
	mTICI ≥ 2b	208.28	409.18	−221.12	637.69	
	Total	63.10	175.66	6.92	119.28	
ShortRunLowGrayLevelEmphasis	mTICI < 2b	0.24	0.16	0.18	0.30	0.043
	mTICI ≥ 2b	0.10	0.05	0.05	0.15	
	Total	0.22	0.16	0.17	0.27	
LargeAreaHighGrayLevelEmphasis	mTICI < 2b	114,139.37	101,830.66	78,608.98	149,669.77	0.014
	mTICI ≥ 2b	245,059.17	174,938.46	614,72.51	428,645.82	
	Total	133,777.34	122,225.54	94,687.72	172,866.97	
Procedure duration (min)	mTICI < 2b	78.79	45.43	62.94	94.64	0.033
	mTICI ≥ 2b	36.67	19.29	16.42	56.91	
	Total	72.48	45.01	58.08	86.87	

**Table 6:** The receiver operating characteristic analysis results of the association between textural features, procedure duration and outcome of mechanical thrombectomy procedure.

Parameter	AUC	Cut-Off	Se (%)	Sp (%)	*p* Value
ClusterProminence	0.768	>3.49	83.3%	69.7%	0.039
ClusterTendency	0.788	>1.11	83.3%	64.6%	0.027
Idmn	0.833	>0.97	97.2%	77.7%	0.010
Idn	0.783	>0.92	97.1%	68.6%	0.029
SumSquares	0.768	>0.3	98.2%	46.5%	0.039
DependenceVariance	0.833	>25.69	99.1%	68.6%	0.010
GrayLevelVariance	0.773	>0.47	83.3%	73.7%	0.036
HighGrayLevelEmphasis	0.859	>5.16	83.3%	64.6%	0.006
LargeDependenceHighGrayLevelEmphasis	0.869	>1291.19	87.3%	79.8%	0.004
GrayLevelVariance	0.783	>0.47	83.3%	73.7%	0.029
HighGrayLevelZoneEmphasis	0.722	>5.16	83.3%	64.6%	0.047
LongRunHighGrayLevelEmphasis	0.838	>36.06	83.3%	77.7%	0.009
LargeAreaHighGrayLevelEmphasis	0.737	>314,525.03	50%	99.4%	0.037
SmallDependenceLowGrayLevelEmphasis	0.793	<0.006	97.9%	77.7%	0.024
ShortRunLowGrayLevelEmphasis	0.813	<0.137	81.8%	83.3%	0.016
Procedure duration	0.785	<34.5	87.9%	77.7%	0.028

**Table 7:** Statistical analysis of the textural features with the outcome of patients on whom a combined thrombectomy procedure was performed.

		Mean	Std. Deviation	95% Confidence Interval for Mean		*p* Value
				Lower Bound	Upper Bound	
ldmn	mTICI < 2b	0.98	0.01	0.97	0.98	0 < 0.001
	mTICI ≥ 2b	0.96	0.02	0.94	0.97	
	Total	0.97	0.02	0.97	0.98	
ldn	mTICI < 2b	0.93	0.14	0.71	0.81	0.004
	mTICI ≥ 2b	0.90	0.11	0.67	0.83	
	Total	0.92	0.13	0.72	0.80	
SmallAreaEmphasis	mTICI < 2b	0.48	0.18	0.42	0.55	0.02
	mTICI ≥ 2b	0.32	0.19	0.18	0.45	
	Total	0.44	0.19	0.38	0.50	

**Table 8:** The receiver operating characteristic analysis results of the association between textural features and outcome of mechanical thrombectomy procedure.

Parameter	AUC	Cut-Off	Se (%)	Sp (%)	*p* Value
Idmn	0.790	<0.97	69%	80%	0.007
Idn	0.814	<0.91	79.3%	80%	0.003
SmallAreaEmphasis	0.772	<0.37	82.8%	70%	0.011

## Data Availability

The data presented in this study are available on request from the corresponding author. The data are not publicly available due to ethical restrictions.
